# Our School System

**Published:** 1879-10

**Authors:** 


### OUR SCHOOL SYSTEM.

It may be safely said, that three teachers out of four do not know the main object of children being sent to school. It is quite as safe to say, that not one school in ten is conducted properly. In not one school in ten are the proper things taught. The chief object in sending children to school is not to give them information, intellectually considered, but to discipline the mind to think for itself ; real information is to be acquired after school days are ended, by this self-thinking, and comparing, and judging. The pert folly, the self-conceit of boys and girls about eighteen, when they begin to think of leaving school, is proverbial. Young people of that age know everything, nothing is doubtful to them ; their poor old parents know nothing, in their estimation, and are regarded with a kind of pitying contempt. "Father," said one of these just from college, "I can prove to you that there are three chickens on the table ; you said there were two." "How do you make it out, my son?" "Why, it is ever so easy, father. This is one chicken, and that is two ; and don't one and two make three ?" "That's a fact, my son. I will take number one, let your mother have number two, and you can have the third one. I hope it will be tender and easily digested, and no doubt you will have a good appetite for breakfast, for I don't intend you shall eat anything else for supper but that third chicken."

A child is educated only in proportion as he learns to think things out himself; and it is from the failure to come up to this point, that we often find to our sorrow that our children know things "by rote," by memory, and are only mechanical scholars.

Three hours in the forenoon, two hours' interval, and two hours more study in the afternoon, and not a moment's study of lessons except in the school-room : let all the other time of the waking existence of each day be expended in muscular activities, in moderate work or recreation involving motion of some kind. No child should be sent to school unless the Kindergarten, before seven years of age, and up to eighteen no child ought to be allowed to continue at brain work, involving intellectual labor, longer than the five hours named.

Up to eighteen years, every child should be allowed ten hours to be in bed. They may not require ten hours' sleep, but time should be allowed to rest in bed, after the sleep is over, until they feel as if they had rather get up than not. It is a very great and mischievous mistake for any person, old or young, especially children, and feeble or sedentary persons, to bounce out of bed the moment they wake up ; all our instincts shrink from it, and fiercely kick against it. Fifteen or twenty minutes spent in gradually waking up, after the eyes are opened, and in turning over and stretching the limbs, does as much good as sound sleep, because these operations set the blood in motion by degrees, tending to equalize the circulation.

